# Simulating Knee-Stress Distribution Using a Computed Tomography-Based Finite Element Model: A Case Study

**DOI:** 10.3390/jfmk8010015

**Published:** 2023-01-27

**Authors:** Kunihiro Watanabe, Hirotaka Mutsuzaki, Takashi Fukaya, Toshiyuki Aoyama, Syuichi Nakajima, Norio Sekine, Koichi Mori

**Affiliations:** 1 Department of Radiology, Shin-Oyama City Hospital, Oyama-shi 323-0827, Tochigi, Japan; 2 Center for Medical Sciences, Faculty of Health Sciences, Ibaraki Prefectural University of Health Sciences, Ami 300-0394, Ibaraki, Japan; 3 Department of Orthopedic Surgery, Ibaraki Prefectural University of Health Sciences Hospital, Ami 300-0331, Ibaraki, Japan; 4 Department of Physical Therapy, Faculty of Health Sciences, Tsukuba International University, Tsuchiura 300-0051, Ibaraki, Japan; 5 Department of Physical Therapy, Ibaraki Prefectural University of Health Sciences, Ami 300-0394, Ibaraki, Japan; 6 Department of Radiological Sciences, Ibaraki Prefectural University of Health Sciences, Ami 300-0394, Ibaraki, Japan; 7 Department of Radiological Sciences, Graduate School of Human Health Sciences, Tokyo Metropolitan University, Arakawa 116-8551, Tokyo, Japan

**Keywords:** osteoarthritis, computed tomography-based finite element method, gait analysis

## Abstract

This study aimed to evaluate the mechanism of progression involved in knee osteoarthritis (OA). We used the computed tomography-based finite element method (CT-FEM) of quantitative X-ray CT imaging to calculate and create a model of the load response phase, wherein the greatest burden is placed on the knee joint while walking. Weight gain was simulated by asking a male individual with a normal gait to carry sandbags on both shoulders. We developed a CT-FEM model that incorporated walking characteristics of individuals. Upon simulating changes owing to a weight gain of approximately 20%, the equivalent stress increased extensively in both medial and lower leg aspects of the femur and increased medio-posteriorly by approximately 230%. As the varus angle increased, stress on the surface of the femoral cartilage did not change significantly. However, the equivalent stress on the surface of the subchondral femur was distributed over a wider area, increasing by approximately 170% in the medio-posterior direction. The range of equivalent stress affecting the lower-leg end of the knee joint widened, and stress on the posterior medial side also increased significantly. It was reconfirmed that weight gain and varus enhancement increase knee-joint stress and cause the progression of OA.

## 1. Introduction

The finite element method (FEM) is a form of structural analysis that has been developed to test the strengths of materials and architectures. Currently, it is used as a technique for analyzing the responses of buildings to external forces [1,2].

In medical imaging technology, the practical application of multidetector row computed tomography (CT) has made it possible to capture isotropic images with three-dimensional equal spatial resolution. By applying FEM technology to the captured image, the bone strength and stress distribution within the joints can be predicted. This technique is called the computed-tomography-based finite element method (CT-FEM) and utilizes quantitative X-ray CT images; it is used clinically—specifically, in orthopedics [3,4]. By using these images, it is possible to predict and evaluate an individual bone’s strength using a three-dimensional bone model for a specific individual [5]. Regarding the CT-FEM models of the knee-joint area, research on joints that consider bones, ligaments, and menisci is slowly progressing [6,7]. The calculation model for predicting intra-articular stress and strain during the load response (LR) phase, wherein the greatest load is applied to the knee joint during walking, is complicated. Osteoarthritis (OA) is the most common knee-joint disease in middle-aged and elderly people [8,9]. OA is a classic example of joint destruction and osteoproliferative changes influenced by significant risk factors for joint degeneration, such as obesity, malalignment, trauma, or changes in joint loading associated with joint instability [10,11,12]. It is an age-related disease, and weight gain in particular causes significant progression of cartilage and meniscal lesions. Aging and mechanical factors are exacerbating factors preceding systemic, genetic, or intrinsic cartilage damage, thereby resulting in the rupture of collagen fibers and eventual destruction of the cartilage matrix. Risk factors for varus deformity in knee OA include age, sex (females carry greater risk), weight, and varus deformity [13]. We have previously constructed a completely new model that combined CT-FEM and motion analysis for knee joints during the LR phase [14,15]. While that study targeted severe knee OA, the processes leading up to severe OA and the factors that increase intra-articular stress could not be evaluated. Weight gain and knee varus are believed to be closely related to OA lesions in the LR phase, which places the greatest burden on the knee joint during walking. We hypothesized that increases in body weight and varus angle would increase stress on the medial side of the knee joint, which is closely associated with knee OA. In this study, we aimed to integrate CT-FEM and motion analysis in order to visualize the impacts of weight gain and variations in the knee varus angle changes on the OA model, as these factors are considered to be closely related to OA lesions. In addition, we also aimed to clarify the involvement of intra-articular stress in aggravation of knee OA.

## 2. Materials and Methods

### 2.1. Participants

A mathematical model was constructed using CT images of the right knee joint of a healthy 43-year-old male with no history of knee OA or surgery involving the knee joint. To create a mathematical model for CT-FEM, the amount of load applied to the right knee joint and the knee flexion angle were based on data obtained from gait analysis measurements. The mathematical method used static linear analysis. This study was conducted in accordance with the Declaration of Helsinki and approved by the Ibaraki Prefectural University of Health Sciences Ethical Review Board (No. e199; date of approval: 27 February 2019). Informed consent was obtained from the participant.

### 2.2. Overview of the CT-FEM Software

**Figure 1:**
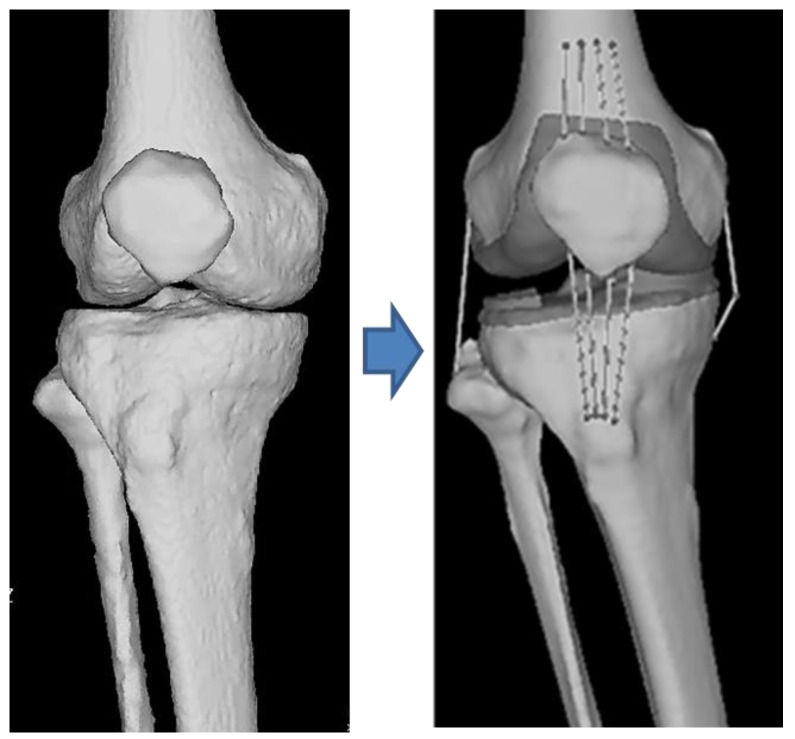
This image is a 3D image obtained by multi-slice CT with ligament, meniscus, and cartilage generated by software.

Imaging of the human body with a multi-slice CT system provides isotropic images of bones. This helps create precise stereoscopic images with the same number of pixels in X, Y, and Z directions. It is possible to obtain the bone density from the CT value of a minute area. Using CT-FEM software, we converted CT images of knee joints (femur, tibia, fibula, and patella) into a three-dimensional model (Figure 1). Joint motion analysis was determined using the Mechanical Finder extended edition: EE version 11.0 (Computational Dynamics Center, Tokyo, Japan). While modeling the knee joint, the femur, patella, fibula, tibia, the ligaments, the menisci, and the main muscles (extensor and flexor muscles) near the knee joint, were defined.

### 2.3. Calibration of CT Image Acquisition Conditions and CT Values

The imaging range was from the center of the thigh diaphysis to the ankle joint. The imaging conditions were as follows: tube voltage, 120 kV; tube current, 175 mA; pitch factor, 0.875; and slice thickness, 0.625 mm [3,7]. An image reconstruction filter was used for the bone standard. Imaging was performed using an eight-row CT device, ECLOS (Hitachi, Ltd., Tokyo, Japan). For CT calibration, a plate-shaped bone mineral quantification phantom, B-MAS200 (Kyoto Science Co., Ltd., Kyoto, Japan), was placed under both lower limbs and was photographed. This made it possible to calibrate fluctuations in CT values that accompany imaging; hence, we could calculate the density of each element of every bone with high reproducibility [7].

### 2.4. Outline up to CT-FEM Analysis

The extended joint position, obtained by extracting the right knee from the CT image, was converted into a flexion angle image obtained from the gait analysis in the LR phase. For walking, a motion capture system (Vicon Nexus software; Oxford Metrics Group, Oxford, UK) and a floor reaction force meter (Kistler Instruments, Winterthur, Switzerland) were used to create the FEM model. Furthermore, to represent obesity and weight gain, an FEM model was created from the gait analysis experiments. Specifically, we modeled (1) walking with his natural body (no overload: NO) and (2) walking with sandbags on both shoulders to simulate weight gain (with overload: WO). Next, we used and modified the right knee model with no increase in the NO + varus angle; four CT-FEM models were created with the femoral side tilted over the knee such that the knee varus angle increased by 5° or 10°, respectively. For each model, the equivalent stress on the femoral cartilage, subchondral femoral joint, tibial cartilage, and subchondral tibial joint surface were computed. Then, an equivalent stress diagram was created. The equivalent stress indicates the absolute stress value when the multiaxial stress state in the three-dimensional space is regarded as a single axis regardless of compression and tension. This was calculated using the von Mises stress formula.

### 2.5. Loading Method

The participant was a male desk worker with a height of 178 cm, a body weight of 78 kg, and a BMI of 23.7. The participant was asked to carry an 8 kg sandbag on each shoulder (total additional weight, 16 kg) while walking. This weight was the maximum load that would not interfere with the pointer used in the gait analysis. The magnitude and direction of the floor’s reaction force during the LR phase were measured using a reaction force meter.

### 2.6. Gait Analysis

From the data obtained by the motion capture system and floor reaction force meter, the walking stance phase was defined according to the phase division suggested by Perry et al. [16]. In the LR phase, when the load on the knee joint was maximized, the angle between the lower limb bone and the floor, angle between the femur and the tibia, muscle strength, segment length, and direction of the external force were reflected in the FEM model for analysis. Table 1 presents the NO and WO values. For varus angle deformity, the femur was tilted 5° or 10° in the varus direction with respect to the angle between the femur and tibia (4.61°), and FEM models were created at 9.61° and 14.61°.

### 2.7. The CT-FEM Model

#### 2.7.1. Scope of Modeling

When setting the analysis range, the upper side was the distal end of the femur, approximately 10 cm from the knee joint, and the lower side was the distal end of the tibia and fibula, just above the ankle joint. The analysis range of each data set was fixed at the same imaging range as the three-dimensional image created by the CT-FEM software.

#### 2.7.2. Construction of the CT-FEM Model

The model was created with reference to a previous study [14]. To construct the CT-FEM model, a tetrahedral solid element (length of one side: 1.0–2.0 mm) was used for the bone part. Furthermore, a triangular-plate-shaped shell element was used to cover the surface of the patella. The size ranges recommended by previous reports were adapted as the sizes of the solid and shell elements [17,18].

Soft tissue elements around the knee, specifically the quadriceps femoris, biceps femoris, semimembranosus, semitendinosus, and gracilis muscles, were set as muscles providing tension around the knee joint during the LR phase of walking. Ligament models of the patella, anterior and posterior cruciate ligaments, and medial and lateral collateral ligaments were made. These were created using Metasequoia (Tetraface Co., Ltd., Tokyo, Japan), which is a three-dimensional modeling software. Each ligament was modeled so that tension was generated only with respect to elongation by using a truss element (line element). Finally, the surface of the patella was covered with a shell element.

#### 2.7.3. Determining Material Properties

For material properties of the bone elements, Keyak’s conversion formula, which has been used often in previous studies, was used for these experiments [19,20,21]. For formulae related to other material properties, we referred to a previous study and to the Poisson’s ratio of 0.4, which is a representative value for bone [22]. Table 2 shows the physical properties of the tetrahedral elements used for the anatomical modeling.

#### 2.7.4. Loading and Restraint Conditions

The load and restraint surfaces were set for the mathematical model we constructed. Regarding the setting of constraint conditions, the femoral epiphysis was completely constrained, and the lower limb epiphysis was constrained along the XY axis to stabilize FEM calculations. The X-axis indicated the left–right component (+: outward direction), the Y-axis indicated the front–back direction (+: forward direction), and the Z-axis indicated the vertical component (+: upward direction). Therefore, this model was used to evaluate the stress generated on the knee joint owing to the component of the floor reaction force parallel to the Z-axis [23].

**Figure 2:**
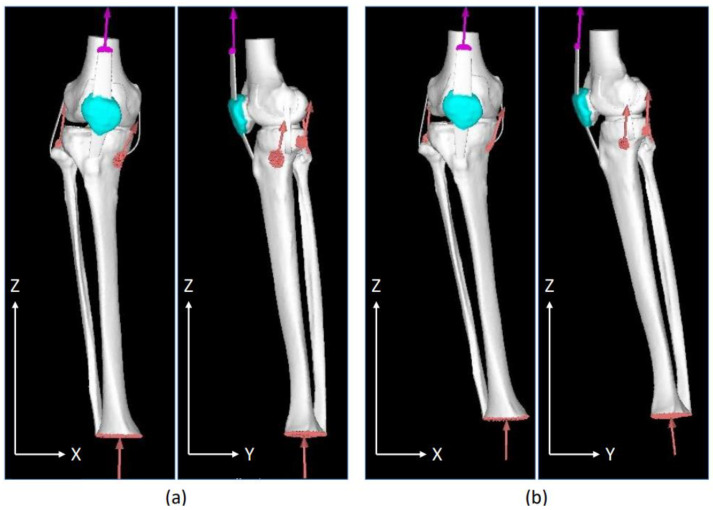
Front and side views of the walking posture and load restraint conditions during the load response phase in a healthy individual. (**a**) No overload; (**b**) with overload.

Quadriceps femoris tension (muscle exertion force) was applied using the forced displacement method. A 2 mm forced displacement was applied as the amount of displacement, with which the same tension as the calculated muscle exertion force could be applied to the truss element. Muscle exertion force was calculated using the musculoskeletal modeling system (Any Body Technology, Aalborg, Denmark) with reference to the values obtained by an optical motion capture system using eight cameras and the floor reaction force meter. The values obtained are listed in Table 3. Contact conditions were set between the femur and tibial cartilage and between the femur and patella cartilage. The coefficient of friction in the contact analysis was set to 0.01 [24,25]. Figure 2 presents the NO and WO models with both loading and restraint conditions. Bone elements, menisci, and articular cartilage are white. The truss element ligaments are colored purple, and both the muscle traction and ground reaction forces are colored orange.

## 3. Results

**Figure 3:**
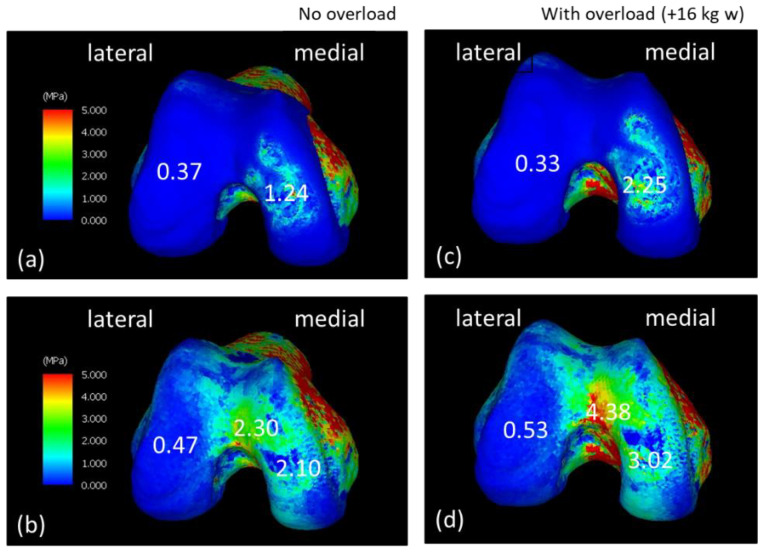
Equivalent stress diagrams of the normal and load states on the femoral end in the load response period during walking. (**a**,**c**) Femoral cartilage surface. (**b**,**d**) Subchondral femoral surface. (**c**,**d**) Equivalent stress diagram under load.

**Figure 4:**
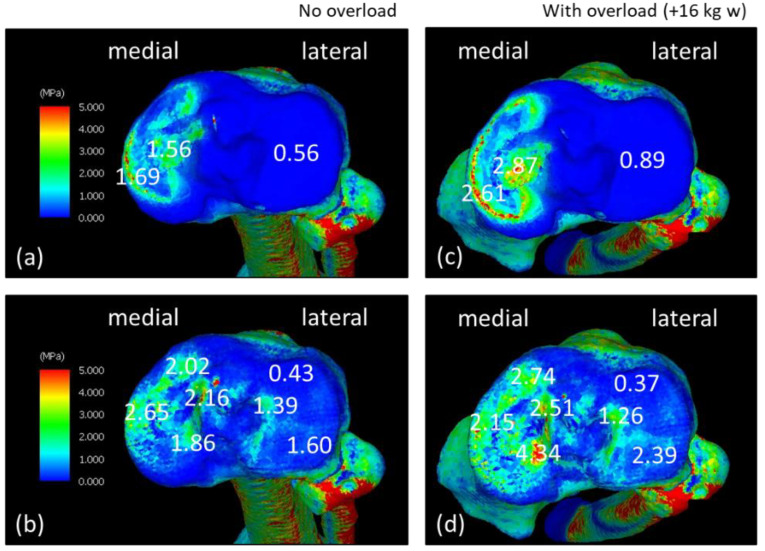
Equivalent stress diagrams of normal and load states on the lower-leg end in the load response phase during walking. (**a**,**c**) Tibial cartilage surface. (**b**,**d**) Subchondral tibial surface. (**c**,**d**) Equivalent stress diagram under load.

Figure 3 shows the equivalent stress diagram on the femoral end during the LR phase while walking. The range of stress values in the equivalent stress diagram was 0–5 MPa. Stress was displayed in order of blue, green, yellow, and red, ranging from the lowest stress to the highest stress. Figure 3a,b show the equivalent stress diagram applied to the femoral and subchondral femoral surfaces of the NO model. Figure 3c,d show the equivalent stress diagram applied to the femoral and subchondral femoral surfaces of the WO model. The equivalent stress on the medial side of the WO model increased, especially on the subchondral femoral surface; the equivalent stress increased widely from the center to the medial side and further anteriorly. Figure 4 shows the equivalent stress on the lower-leg end during the LR phase while walking. Figure 4a,b show the equivalent stress diagram applied to the tibial cartilage and subchondral tibial surfaces of the NO model. Figure 4c,d show an equivalent stress diagram applied to the tibial and subchondral tibial surfaces of the WO model. The tibial cartilage surface increased in equivalent stress over a large area, mainly behind the medial–lateral margin owing to weight gain, and the equivalent stress increased by approximately 150% in the margin. Stress on the subchondral tibial surface increased considerably and was dispersed over a wide area on the medial side. The stress on the lateral side also increased. The stress increased by approximately 230% from 1.86 to 4.34 MPa behind the medial side.

**Figure 5:**
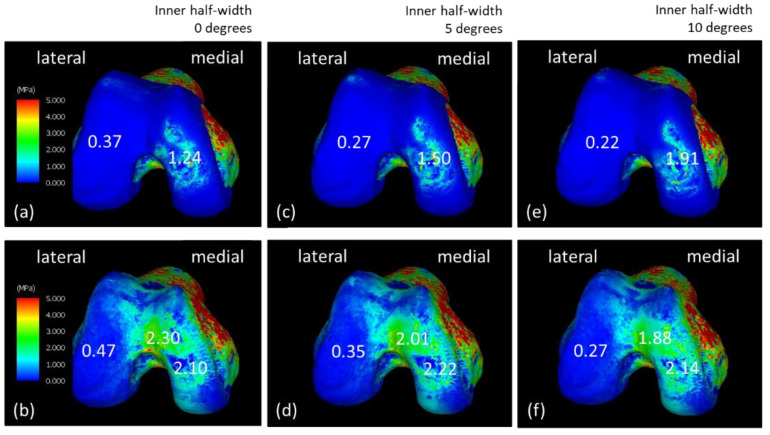
Equivalent stress diagrams of the normal state of the femoral end and changes in the inner-half’s angle during the load response phase of walking. (**a**,**c**,**e**) Femoral cartilage and (**b**,**d**,**f**) subchondral femoral surfaces. (**c**,**d**) The varus angle increased by 5° and (**e**,**f**) 10°.

Figure 5 presents the equivalent stress on the femoral end when the varus angle of the knee increased. Figure 5a,b show equivalent stress of the femoral cartilage and subchondral femoral surfaces during walking, respectively; Figure 5c,d show the femoral cartilage surface and that cartilage with a varus angle of +5 degrees. They show equivalent stress on the surface of the lower femur. Equivalent stress on the surface of the femoral cartilage with a varus angle of +10 degrees is shown in Figure 5e, and equivalent stress on the surface of the subchondral femur is presented in Figure 5f.

**Figure 6:**
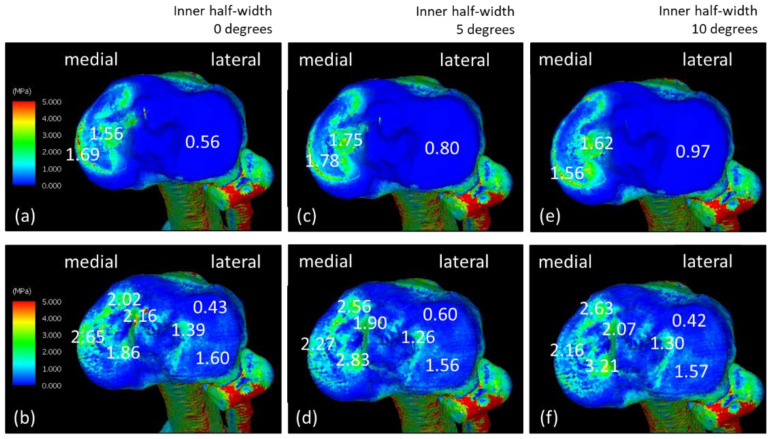
Equivalent stress diagrams of the bone side of the lower leg in normal state and change in the inner-half angle’s during the load response period during walking. (**a**,**c**,**e**) Tibial cartilage and (**b**,**d**,**f**) subchondral tibial surfaces. (**c**,**d**) The varus angle increased by 5° or (**e**,**f**) 10°.

Figure 6 shows equivalent stress on the lower-leg end when the angle of the knee varus is changed. Figure 6a,b show equivalent stress on the tibial cartilage and subchondral tibial surface during walking. Figure 6c,d show equivalent stresses on the tibial cartilage and subchondral tibial surfaces with a varus angle of +5°. Equivalent stress on the tibial cartilage surface with a varus angle +10° is shown in Figure 6e, and equivalent stress on the subchondral tibial surface is shown in Figure 6f. The varus angle tilted the femoral side from 0° to 10°. However, the stress change on the femoral side cartilage surface was small, and the subchondral femoral surface area of the thigh expanded in the lateral center. On the lower-leg end, the stress was higher (increased from 1.86 to 3.21 MPa), especially on the posteromedial side, and the amount of stress on the entire medial side increased on both the tibial cartilage and the subchondral tibial surface.

## 4. Discussion

We constructed a knee joint CT-FEM model that incorporates an increase in both weight and knee varus angle during the LR phase while walking, followed by investigation of equivalent stress on the femoral end and the lower-leg end of the knee joint. To strengthen the calculation of the moment generated by the muscle traction and floor reaction forces, this analysis was performed while constraining the XY direction of the distal radius. The horizontal component in the XY direction was expected to be approximately 20% of the vertical component [26]. Therefore, only the effect of normal stress on the joint was calculated. Interestingly, the main effect observed was related to the floor reaction force.

At the stress level of the ground reaction force due to the vertical component, the stress was strongly applied over a wide area from the center of the medial condyle, which was inside the joint. In a normal knee, the arc of the medial femoral condyle is maintained so that force can be distributed and received by both the meniscus and the articular cartilage. In addition, any change due to stress has a greater effect on the lower-leg end than the femoral end [27,28]. This indicates that the joint reaction forces, which include the deformation and damage to the joint due to a resultant force, such as floor reaction and muscle toning, may occur over the meniscus and the rough surface of the tibia. The area of high stress near the medial center of the tibial cartilage surface is where the cartilage is in direct contact with other cartilages, and it is known to be vulnerable to loading stress [29]. The difference between subchondral bone and cartilage is assumed to be that the high-stress areas of cartilage are localized and actually touching. The function of articular cartilage is to spread the load as a shock absorber that supports large amounts of weight while preventing friction at the epiphysis. The stress generated locally in the cartilage spreads over a wide area in the subchondral bone, possibly because the stress is dispersed and the force is applied to a wide range of bones, resulting in a different image for cartilage.

Using data from healthy individuals, greater stress was seen to be applied to the medial surface on both the femoral and tibial ends as an effect of weight gain. Although the data obtained during the actual walking experiment were used in this calculation, it was necessary to consider the influences of the internal rotation and flexion angles in addition to the stress load generated within the joint. The weight gain using the sandbags was 16 kg; however, the concomitant increase in the floor reaction force was approximately 24 kg. This is the effect of the mechanical action on the lower extremity induced by body weight and locomotion. According to this calculation model, a stronger varus angle results in lower lateral stress on the joint and higher stress on the medial rear part of the joint. This is consistent with clinical findings and complaints of pain in patients with knee OA [30]. Therefore, in a model that combines CT-FEM and gait analysis, we have herein reconfirmed that weight gain and varus angle enhancement increase stress on the medial side of the joint and pose an OA risk. Hence, this model may be a means to infer the mechanism of gait and how it changes during OA progression. Cross-sectional epidemiological studies have pointed out that weight gain and knee varus are risk factors for knee OA progression [31]. Furthermore, a computational model simulating an increase in the coefficient of friction between the meniscus and the articular cartilage, along with a computational model with an internal rotation angle, may help predict the progression from a healthy to an OA knee [32,33]. Being able to image changes in weight gain and varus angle allowed us to see how much stress would change the actual knee joint. The ultimate goal is to prevent progression of a normal knee to OA, and in the future, we will evaluate it from a biomechanical point of view, and it will be useful for walking modifications.

The most important limitation of this study was the presence of only one participant. In general, knee OA is more common among older women. Therefore, in future, a large-scale study similar to the present one, including older women, is needed. However, not all patients exhibit similar progression to OA because each patient has a different walking style and lifestyle. It is also important to analyze the gait of each individual and investigate the process by which each individual progresses to OA. In this study, we created an FEM model using CT images of the knees of a healthy individual. Furthermore, it is difficult to perform mechanical tests to confirm the predictive results of CT-FEM, and these tests must be performed on cadavers.

## 5. Conclusions

The equivalent stress value arising from an increase in the floor reaction force due to weight gain was calculated using this FEM model. Furthermore, an additional equivalent stress value was obtained using a simulation, in which the varus angle of the knee was changed. Increased weight gain and knee varus angle increased intra-articular stress on both the distal medial femur and proximal medial tibia. By combining a motion analyzer, reaction force meter on a platform, and muscle-traction-force calculation, a more accurate CT-FEM model can be constructed.

Clinically, weight gain and increased varus angle are well known risk factors for knee osteoarthritis [34,35]. Investigating how the weight gain and changes in the varus angle in this study individually affect the stress on the knee joint will be useful for understanding the onset and progression of knee OA. This is considered to be a suggestion for devising preventive measures.

## Figures and Tables

**Table 1:** The angles between the femur and tibia and the tibia and floor.

	Angle between the tibia and floor		
No overload (NO)	Varus angle 6.18°	Flexion angle 10.42°	Internal rotation 41.26°
With overload (WO)	Varus angle 5.86°	Flexion angle −0.27°	Internal rotation 44.84°
	Angle between the femur and tibia		
No overload (NO)	Varus angle 4.61°	Flexion angle 2.30°	Internal rotation 0°
With overload (WO)	Varus angle 6.80°	Flexion angle 7.90°	Internal rotation 0°

**Table 2:** Material properties of the simulated anatomical elements around the knee.

Anatomical Element	Poisson’s Ratio	Young’s Modulus (MPa)
Femur, tibia, fibula, patella	0.4	Keyak’s conversion formula
Cartilage	0.4	20 (100 only on the fibula)
Meniscal	0.4	20
Ligament	0.4	0.1

**Table 3:** The measured floor reaction force and calculated traction force of each simulated muscle.

Muscle Traction (N)		
	No Overload (NO)	With Overload (WO)
Quadriceps	392.49	608.51
Biceps femoris	405.24	620.51
Semimembranosus	166.13	245.96
Semitendinosus + gracilis	96.48	133.30
Floor reaction force (N)		
	No overload (NO)	With overload (WO)
Inward direction	47.67	43.30
Forward direction	43.97	67.52
Upward direction	753.44	987.42

## Data Availability

Not applicable.
